# The Association between Iron and Vitamin D Status in Female Elite Athletes

**DOI:** 10.3390/nu10020167

**Published:** 2018-01-31

**Authors:** Jadwiga Malczewska-Lenczowska, Dariusz Sitkowski, Olga Surała, Joanna Orysiak, Beata Szczepańska, Konrad Witek

**Affiliations:** 1 Department of Nutrition Physiology and Dietetics, Institute of Sport, National Research Institute, Trylogii 2/16, 01-982 Warsaw, Poland; olga.surala@insp.waw.pl (O.S.); joanna.orysiak@insp.waw.pl (J.O.); beata.szczepanska@insp.waw.pl (B.S.); 2 Department of Physiology, Institute of Sport, National Research Institute, Trylogii 2/16, 01-982 Warsaw, Poland; dariusz.sitkowski@insp.waw.pl; 3 Department of Biochemistry, Institute of Sport, National Research Institute, Trylogii 2/16, 01-982 Warsaw, Poland; konrad.witek@insp.waw.pl

**Keywords:** iron status, 25(OH)D, vitamin D status, mutual relationships, healthy female, athletes

## Abstract

Vitamin D may influence iron metabolism and erythropoiesis, whereas iron is essential for vitamin D synthesis. We examined whether vitamin D deficiencies (VDD) are associated with reduced iron status and whether progressive iron deficiency (ID) is accompanied by inferior vitamin D status. The study included 219 healthy female (14–34 years old) athletes. VDD was defined as a 25(OH)D concentration < 75 nmol/L. ID was classified based on ferritin, soluble transferrin receptor (sTfR), total iron binding capacity (TIBC) and blood morphology indices. The percentage of ID subjects was higher (32%) in the VDD group than in the 25(OH)D sufficient group (11%) (χ^2^ = 10.6; *p* = 0.001). The percentage of VDD subjects was higher (75%) in the ID than in the normal iron status group (48%) (χ^2^ = 15.6; *p* = 0.001). The odds ratios (ORs) for VDD increased from 1.75 (95% CI 1.02–2.99; *p* = 0.040) to 4.6 (95% CI 1.81–11.65; *p* = 0.001) with progressing iron deficiency. ID was dependent on VDD in both VDD groups (25(OH)D < 75 and < 50 nmol/L). The ID group had a lower 25(OH)D concentration (*p* = 0.000). The VDD group had lower ferritin (*p* = 0.043) and iron (*p* = 0.004) concentrations and higher values of TIBC (*p* = 0.016) and sTfR (*p* = 0.001). The current results confirm the association between vitamin D and iron status in female athletes, although it is difficult to assess exactly which of these nutrients exerts a stronger influence over the other.

## 1. Introduction

Iron and vitamin D are two essential nutrients which constitute an important worldwide health issue due to their significant roles in biochemistry and simultaneously, the very high risk of deficiency in both of them [1,2].

Vitamin D plays a dual role in the human body as a prohormone nutrient and fat soluble vitamin. Due to its pleiotropic nature, beyond its influence on bone health, vitamin D demonstrates significant involvement in various gene expression processes and plays key roles in calcium and phosphate metabolism, which are involved in a multitude of physiological and pathophysiological mechanisms [3]. Deficiency in vitamin D is linked to numerous illnesses and pathological conditions, including musculoskeletal health, immunity, cardiovascular disease, cancer and mental health [4], as well as deterioration of athletic performance [5,6,7]. The high prevalence of low serum vitamin D concentration is a global problem in all age groups, even in regions of high sun exposure [8]. Athletes appear to have a similar risk of vitamin D deficiency as nonathletic subjects from the same population. Seasonal variance in vitamin D status is observed in athletes as well as in the general population [9,10]. However, it should be noted that exercise-induced stress may also promote deterioration of vitamin D levels, especially in athletes training and competing indoors [5,6,7,11,12].

Iron is another essential nutrient which is involved in many physiological processes, particularly in the production of red blood cells and myoglobin, oxygen transport and the production of ATP, DNA synthesis, and electron transport in mitochondria [1,13,14]. Although the human system has created mechanisms for preventing iron deficiency, the lack of this mineral is one of the basic factors associated with anemia [15]. Approximately 50% of all anemia cases in developed countries are caused by iron deficiency [2]. The groups particularly exposed to deficiencies of this mineral are women of reproductive age, children and adolescents [2,16,17]. Results from many studies indicate that athletes are also at high risk for iron deficiency [18,19,20], and this applies especially to physically active women [19,21,22].

Numerous cross-sectional studies have indicated an association between low 25(OH)D concentration and poor iron status [23,24,25,26]. Furthermore, Azizi-Soleiman [1], in a systematic review, pointed out that such relationships may be mutual. It is known that a deficit of vitamin D may cause deterioration of iron status [27,28] and increase the risk of anemia [26,29,30]. The precise mechanisms for this dependence are still not understood [26], but it is hypothesized that vitamin D may affect iron regulation and erythropoiesis by its influence on hepcidin via cytokines [31,32] or independently of changes in pro-inflammatory markers [33,34]. There are also findings indicating that vitamin D may directly influence erythroid precursors in bone marrow [23,35].

Iron deficiency, in turn, was identified as one of the factors for vitamin D deficiency. The positive correlation between these two nutrients is confirmed by an increase of vitamin D concentration after intramuscular iron treatment in infants [36] as well as a positive correlation between hematological and non-heme indices of iron status with 25(OH)D concentration [24,36,37], although the exact mechanisms of this dependence are also not known. There is evidence that a deficit in iron may disturb the synthesis of vitamin D_3_ and lead to its mild deficiency, because conversion of cholecalciferol to the biologically active form, calcitriol (1,25-dihydroxyvitamin D_3_) requires two steps of hydroxylation—the first in the liver and the second in the kidney—which depend on enzymes containing heme, i.e., cytochromes P450 (CYP2R1 and CYP27B1 respectively) [38,39].

Both nutrients have frequently drawn the attention of researchers, and there is a wealth of data on athletes concerning either the assessment of vitamin D [10,12,40] or iron status in athletes [19,40,41]; however, there are still few studies examining the interdependence between them. So far, only Constantini et al. [27] have analyzed the relationship between both of these nutritional components. They observed the influence of vitamin D levels on iron and serum ferritin concentrations. Moreover, there is a lack of research investigating the impact of iron on vitamin D status in physically active people. To the best of our knowledge, there is no research in which the mutual relationship between these two nutrients has been examined.

Since female athletes are a group at particularly high risk of both vitamin D and iron deficiency, it seemed sensible to examine: (1) whether deficiencies of vitamin D are associated with reduced iron status and (2) whether progressive iron deficiency is accompanied by inferior vitamin D status.

## 2. Material and Methods

### 2.1. Subjects

Initially, venous blood samples were obtained from 231 female professional athletes, representative of seven sports disciplines: volleyball, handball, rowing, canoe sprint, cycling, speed skating and taekwondo. All of the studied athletes were Caucasian, and most of them were members of the national team. The blood was withdrawn from each subject only once, at various phases of their training cycles and seasons. Fifty one percent of the studied population was examined during period of effective synthesis of vitamin D_3_ (April–September). The subjects were not interviewed regarding iron dietary intake or iron and vitamin D supplementation. All procedures performed in studies involving human participants were in accordance with the ethical standards of the institutional and/or national research committee and with the 1964 Helsinki Declaration and its later amendments or comparable ethical standards. Ethical approval for this study was provided by the local ethical committee at the Institute of Sport—National Research Institute in Warsaw, Poland (protocols: #KEBN-16-25-JO, and #KEBN-15-8-DT). All subjects gave their informed consent for inclusion before they participated in the study. Written informed consent was obtained from participants or their parents if the athletes were under 18 years of age. In order to rule out factors that may have a potential impact on indices of iron status, three exclusion criteria were applied: presence of any symptoms of acute phase reaction, i.e., increased values of erythrocyte sedimentation rate (ESR); C-reactive protein concentration (CRP); or white blood cell count (WBC). Twelve female athletes failed to fulfill the criteria and were subsequently excluded from the study. Finally, 219 females who were found to be healthy were included in the statistical analysis. Basic data concerning the characteristics of the studied subjects are presented in Table 1.

### 2.2. Blood Analysis

The blood was withdrawn from the antecubital vein in the morning (between 8 and 9 a.m.) in the pre-prandial state, after overnight fasting. To eliminate any residual effects of physical movement and ensure the data collected reflected a resting baseline, sample collection started after a minimum 10-min rest, in a seated position. In order to obtain the serum for testing, blood samples were centrifuged for 10 min at a speed of 3500 rpm.

#### 2.2.1. Blood Morphology Indices

In whole blood, the following measurements were performed in mature erythrocytes using the ADVIA 120 hematology system (Siemens Healthcare, Erlangen, Germany): hematocrit (HCT), hemoglobin concentration (Hb), red blood cell count (RBC), mean corpuscular hemoglobin concentration (MCHC), mean corpuscular volume (MCV), mean corpuscular hemoglobin (MCH), mean cellular hemoglobin content (CH), percentage of erythrocytes with decreased cellular hemoglobin concentration (%HYPOm), percentage of erythrocytes with decreased cellular hemoglobin content (%LowCHm), percentage of erythrocytes with decreased volume (%MICROm), and red cell volume distribution width (RDW). In reticulocytes the following parameters were measured: mean cellular hemoglobin content (CHr), reticulocyte count expressed as an absolute number (#RET) and as a percentage of the absolute value (%RET), mean cellular hemoglobin concentration (CHCMr), mean corpuscular volume (MCVr), percentage of reticulocytes with decreased cellular hemoglobin concentration (%HYPOr), and percentage of reticulocyte population with decreased cellular hemoglobin content (%LowCHr). These analyses were done within 3 h of blood collection. Within-run precision of the hematological parameters, expressed as the coefficient of variation (CV), obtained from 20 repetitions of the same blood sample, was as follows: Hb 1.26%; HCT 1.01%; RBC 0.93%; MCHC 0.88%; MCV 0.15%; MCH 0.89%; CH 0.19; RDW 0.98%; %HYPOm 13.8%; %LowCHm 1.72%; %MICROm 6.68%; CHr 0.31%; #RET 5.05%; %RET 5.31%; CHCMr 0.62%; MCVr 0.69%; %HYPOr 21.4% and %LowCHr 21.4%.

#### 2.2.2. Vitamin D and Iron Status Indices in Serum

Total serum 25(OH)D concentration was analyzed using commercially available ELISA kits (DiASource, Louvain-La-Neuve, Belgium), according to the manufacturer’s protocol. All assays were performed in duplicate. The coefficient of variation of the intra-assays for 25(OH)D concentration in this study was below 4%. The 25OH vitamin D Total ELISA kits (DiASource, Louvain-La-Neuve, Belgium) have a Certificate of Proficiency issued by the vitamin D External Quality Assessment Scheme (DEQAS) Advisory Panel. According to the manufacturer’s instructions, concentrations of 25(OH)D below 25 nmol/L were classified as a deficiency, and values of 25–75 nmol/L were considered a vitamin D insufficiency.

The ferritin concentration was measured using the immunoturbidimetric method, enhanced with latex particles. Iron concentration and unsaturated iron binging capacity (UIBC) were determined using spectrophotometric methods with FerroZine. All mentioned indicators were measured on a Roche Cobas Integra 400 biochemical analyzer (Roche Diagnostics, Rotkreuz, Switzerland) using original manufacturer reagent kits. Inter-assay variability for those indices did not exceed 7.8%, 1.3% and 1.7%, respectively. Total iron binding capacity (TIBC) was calculated from iron and UIBC (the sum of both indicators). Soluble transferrin receptor (sTfR) concentration was measured using immunoenzymatic commercial kits (RamcoStafford, TX, USA). Inter-assay variability for this indicator did not exceed 6.0%. All assays were performed in duplicate, in never-frozen or only once-frozen (−20 °C) serum samples.

Each stage of iron deficiency was based on ferritin, sTfR, TIBC and three basic morphological indices: Hb, RBC and HCT. There is no unified consensus threshold value of ferritin for diagnosing iron deficiency. In this study, 16 µg/L was considered to be the lower threshold value [20]. Iron stores were considered low (stage I of iron deficiency, ID) if ferritin concentrations (only) were below 16 µg/L. The basis for the diagnosis of latent iron deficiency (stage II of ID) was elevated sTfR concentrations (>8.3 mg/L), and/or increased TIBC (>390 µg/dL). Iron deficiency anemia (IDA) was diagnosed when a low ferritin concentration and increased values of sTfR and TIBC were accompanied by low values of Hb, HCT and RBC. According to the ADVIA 120 hematology system (Siemens Healthcare, Erlangen, Germany) the following values of these three indices were considered anemic indicators in female subjects: Hb < 120 g/L, HCT < 37% and RBC < 4.2 × 10^12^/L.

#### 2.2.3. Acute Phase Reaction Markers

To assess the acute phase reaction in whole blood the white blood cell count (WBC) (ADVIA 120 hematology system, Siemens Healthcare, Erlangen, Germany), and erythrocyte sedimentation rate (ESR) after one hour were measured. In serum, the C-reactive protein (CRP) concentration was determined by the immunoturbidimetric method, reinforced with latex particles on a Roche Cobas Integra 400 biochemical analyzer (Roche Diagnostics, Rotkreuz, Switzerland) using original manufacturer reagent kits. The CRP reagent used in the study covered a wide range of linearity, containing both normal (1.0–5.0 mg/L) and inflammatory response ranges (5.0–160.0 mg/L). Inter-assay variability for those indices did not exceed 2.9%.

All analyses were performed in a biochemical laboratory with an implemented quality control system.

### 2.3. Anthropometric Measurements

Body height was assessed using a Siber Hegner anthropometer (Zurich, Switzerland) with an accuracy of 0.1 cm. Body mass (BM) was measured using a Tanita MC 180MA instrument (Middlesex, UK), and body composition was estimated by skinfold thickness measurements (Holtain Skinfold Caliper, Crymych, UK) using the four-site formula by Durnin and Womersley [42].

### 2.4. Statistical Analysis

All statistical analyses were conducted with Statistica 13, Dell Inc. (Tulsa, OK, USA). The data are presented as mean ± standard deviation.

The chi-square test was used to compare the frequency of subjects with sufficient or insufficient vitamin D levels in groups with iron deficit and normal iron status as well as the frequency of occurrence of normal iron status and iron deficiency in subjects with reduced and sufficient vitamin D concentrations.

To examine the simultaneous influence of several variables on the risk of deficiency for both vitamin D and iron, multivariate logistic regression analysis (with stepwise procedure) was performed (ORs, 95% CI). In the case of vitamin D deficiency, ORs were adjusted for the following confounding factors: iron deficiency, length of day, in-/outdoor disciplines (dichotomous variables), and age (continuous variable). In iron deficiency, the same confounding factors were used, although vitamin D deficiency was applied in place of iron deficiency.

Unadjusted logistic regression analysis (ORs, 95% CI) was performed in order to assess: (1) the risk of vitamin D deficiency in groups with low iron stores based on different values of ferritin (range from 30 to 12 µg/L) due to the lack of a unified consensus threshold value of ferritin and additionally, in the group with stage II iron deficiency together with IDA; and (2) the risk of iron deficiency in subjects with 25(OH)D concentrations below 25 and 75 nmol/L.

The Mann–Whitney U test was used to compare the mean values of 25(OH)D in groups of athletes with normal iron status and iron deficit as well as to compare the mean values of iron status and blood morphology indices in groups of athletes with a sufficient or insufficient vitamin D level. A value of *p* < 0.05 was considered statistically significant.

## 3. Results

Mean values (±SD) and ranges of all studied indices in the whole group of female athletes are presented in Table 2.

The frequency of female athletes with 25(OH)D concentration below 75 nmol/L was 54.3%, wherein most subjects showed insufficient concentrations (within 25–75 nmol/L). A deficit of this vitamin (<25 nmol/L) was only observed in 1.8% of females.

Total iron deficiency was identified in 23.3% of female athletes. Low iron stores were observed in 7.3%, latent ID in 15.1% and iron deficiency anemia in 0.9% of subjects.

Logistic regression analysis (Table 3), expressed as the odds ratio (OR), with a 95% confidence interval (95%CI), indicated that in female athletes, vitamin D deficiency was significantly (*p* = 0.01) affected by two factors: the length of day (OR = 2.29; 95% CI 1.28–4.07; *p* = 0.005) and iron deficiency (OR = 2.96; 95% CI 1.45–6.02; *p* = 0.003). Iron deficiency, in turn, was correlated with vitamin D deficiency (OR = 2.73; 95% CI 1.32–5.62; *p* = 0.007) and age (OR = 0.82; 95% CI 0.73–0.91; *p* = 0.000).

**Figure 1:**
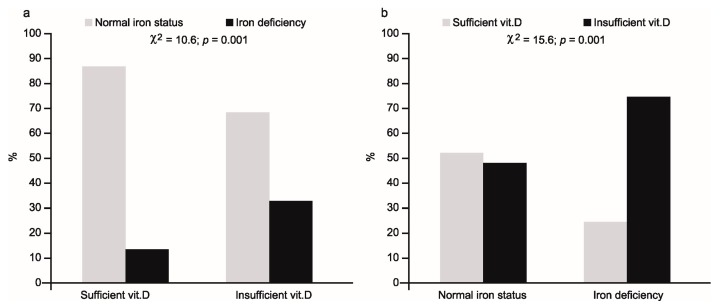
Percentage of athletes with normal iron status or iron deficiency in relation to vitamin D status (**a**) and with sufficient or insufficient concentration of 25(OH)D in relation to iron status (**b**).

The percentage of athletes with iron deficiency and normal iron status in relation to vitamin D status ((a)—left part of figure) and vice versa ((b)—right part of figure) is shown in Figure 1.

In the first case, iron deficiency was present in 32% of 25(OH)D deficient subjects, compared with 11% in the vitamin sufficient group (χ^2^ = 10.6; *p* = 0.001). Conversely, low 25(OH)D concentration was observed in 75% of iron deficient females, compared with 48% of subjects with normal iron status (χ^2^ = 15.6; *p* = 0.001).

The odds ratios for vitamin D deficiency were significantly higher for those with iron deficiency, and the values of ORs increased along with a decreasing level of ferritin as a criterion for iron deficiency, from 1.75 (95% CI 1.02–2.99; *p* = 0.040) at a ferritin level of 30 µg/L to 3.56 (95% CI 1.6–3.12; *p* = 0.002) at a ferritin level of 12 µg/L. The highest value of OR equal to 4.6 (95% CI 1.81–11.65; *p* = 0.001) was observed in the group with more advanced iron deficiency, i.e., in subjects with latent ID and IDA (Table 4).

The ORs for iron deficiency were significantly higher for both the subjects with concentrations of 25(OH)D < 75 nmol/L and for those with concentrations of <50 nmol/L (Table 4). The ORs for those groups were 3.14 (95% CI 1.56–6.31; *p* = 0.001) and 3.18 (95% CI 1.09–9.26; *p* = 0.030), respectively, and did not differ between each other.

In athletes with iron deficiency, significantly lower mean serum 25(OH)D concentrations (*p* = 0.000) were observed (Table 5), while the group with insufficient vitamin D concentrations had significantly different values for all four indices of iron status: ferritin, iron, sTfR and TIBC (Table 6).

The mean values of ferritin (*p* = 0.043) and iron (*p* = 0.004) were significantly lower, while the mean values of TIBC (*p* = 0.016) and sTfR (*p* = 0.001) were significantly higher, compared to the group with normal 25(OH)D concentration (Table 6). Additionally, in the group with reduced vitamin D concentration, lower mean values of some hematological indices in reticulocytes (CHr, *p* = 0.049 and MCVr, *p* = 0.020) were observed, although the mean value of RBC in this group was higher (*p* = 0.029).

## 4. Discussion

The high frequency of both iron and vitamin D deficiencies observed in the present study are in accordance with many earlier studies concerning physically active subjects [10,12,19,21,22,27,43]. This confirms that the problem regarding deficiencies in both nutrients in athletes is still present.

With gradual recognition of the role of vitamin D deficiency in many diseases [4], the relationship between vitamin D and iron status has also begun to be explored [1]. It has been demonstrated that a deficit of vitamin D increases the risk of many hematological disorders and iron metabolism disturbances [26,44], which was visible, especially in adults with different illnesses [26]. One reason for this is the pro-inflammatory effects of a vitamin D deficit, which eventually leads to an increase in hepcidin production, via stimulation of pro-inflammatory cytokines [31] and activation of the JAK-STAT3 pathway [44]. Sun et al. [34] pointed out that vitamin D can also downregulate hepcidin transcription, although the mechanism by which this occurs is unknown. High hepcidin levels, in turn, may favor sequestration of iron in macrophages and hepatocytes, which promotes the development of inflammatory anemia [32]. This anti-inflammatory effect of vitamin D is confirmed by studies pointing to the reduction in hepcidin levels and increase in 25(OH)D concentration in vitamin D deficient subjects after supplementation with this vitamin [33].

The lack of association between vitamin D status and the two main hematology parameters used to diagnose anemia—Hb and HCT—and the higher mean values of RBC, in the group with reduced vitamin D concentrations in our study, may have resulted from the fact that only healthy athletes (without any symptoms of acute phase reaction) were studied. This confirms the hypothesis that vitamin D deficiency might be particularly associated with inflammatory anemia [45], although it is worth emphasizing that a positive association between vitamin D and morphological parameters was also observed in healthy adults [23].

The logistic regression analysis indicated, however, that in female athletes, in addition to age (lower age favors iron deficiency), vitamin D deficiency significantly increases the risk of ID (Table 3). The reverse relationship between age and iron status in females is known [46], but the impact of vitamin D on iron status is less proven, especially in terms of its effect on non-hematological parameters. Despite the association between vitamin D and blood morphology indices having been investigated relatively often, the results are contradictory, mainly due to the study of different subtypes of anemia [47] and presence of inflammation [23]. Furthermore, in some studies, only ferritin was measured [27,33] and non-heme indices were not tested at all [48]. In our study the frequency of iron deficiency was significantly higher in the group with reduced 25(OH)D concentrations (Figure 1). Simultaneously, in this group, we observed significant changes in all four indices of iron status. Positive relationships of ferritin and iron concentrations and negative relationships of TIBC values and sTfR concentrations (Table 6) clearly indicated a worsening of both storage and transport iron pools in the body. In addition, in the group with reduced vitamin D levels, significantly lower values of two (independent of plasma volume) hematological parameters, including reticulocytes, were observed. A lower mean corpuscular volume (MCVr) and mean corpuscular hemoglobin concentration (CHr) in reticulocytes indicates that the deficit of iron in this group had started to affect the functional iron pool as well. This is logical because in the group with vitamin D deficiency, a relatively high percentage (about 76%) of subjects with iron deficiency manifested a more advanced deficit in iron (i.e., stage II iron deficiency) with two athletes having iron deficiency anemia. The observed, simultaneously opposite relationship between vitamin D status and RBC may be due to post-exercise changes in plasma volume [49].

There are several possible mechanisms which could explain the impairment of iron status coexisting with vitamin D deficiency. One of them assumes that insufficient quantities of vitamin D may impair iron availability and its absorption, via an increase in hepcidin concentration, due to the increase in some cytokines—e.g., IL-6 or IL-1B [31,32]—which may also take place after physical effort [50]. However, the results of the recent study by Smith et al. [33] indicated that in healthy adults, vitamin D may act on hepcidin directly—that is, without cytokines. Moreover, hepcidin may not only hamper the availability of iron from monocytes, hepatocytes and enterocytes, through the iron–hepcidin–ferroportin axis [31], but additionally, may impair absorption of iron due to a decrease in duodenal divalent metal transporter 1 (DMT1) levels [51]. Even though we excluded all the subjects with any symptoms of an acute phase response, we unfortunately did not measure hepcidin and proinflammatory cytokine concentrations, which, as a consequence, did not allow for a more accurate analysis of the obtained results. In this situation, we can only presume that hepcidin may be involved in the deterioration of iron status, although a direct effect of vitamin D on red blood cell production is also possible. It has been reported that metabolites of vitamin D (especially its active form) are crucial for normal red blood cell production, via the stimulation of erythroid progenitor cells in a synergistic fashion with erythropoietin [23,35]. In bone marrow, the levels of 25(OH)D and (1,25(OH)_2_D) are 25- and 500-fold higher, respectively, in comparison to serum [52]. Low 25(OH)D levels in marrow tissue may lead to insufficient substrate availability for 1α-hydroxylase-induced synthesis of the active form of vitamin D, which is needed for hematopoiesis [35].

It is worth noting that the risk of iron deficiency did not increase with decreasing 25(OH)D concentration (Table 4). Similar OR values for both 75 and 50 nmol/L concentrations (3.14, 95% CI 1.56–6.31, *p* = 0.001 and 3.18, 95% CI 1.09–9.26, *p* = 0.030, respectively) indicated that a vitamin D concentration below 75 nmol/L may be impacted by worsening of the iron status. Our results are consistent with the results of others. Similar differences in OR values for 50 and 75 nmol/L were observed by Atkinson et al. [48] in white children in the USA. Sim et al. [23] also indicated 75 nmol/L as a limit value, although there are data supporting a lower threshold value (i.e., 50 nmol/L) below which the risk of anemia becomes clearly higher [29].

Whereas the impact of vitamin D deficiency on blood morphology and non-hematological iron status indices has been studied fairly often, the inverse association between these two nutrients has been tested less often. Some studies concerning bone tissue indicate that iron deficiency is a risk factor for both impaired vitamin D and bone metabolism in humans [37,38]. The present results are in line with this relationship, because, in our study, iron deficiency was also related to the worsening of vitamin D status. This was confirmed by the multivariable-adjusted logistic regression model, which indicated iron status (OR = 2.96, 95% CI 1.45–6.02, *p* < 0.003) and length of day (OR = 2.29, 95% CI 1.28–4.07, *p* < 0.005) as the two factors significantly affecting vitamin D status. Furthermore, among iron deficient subjects, the percentage of female athletes with a low 25(OH)D concentration was relatively high (75%)—significantly higher than in the group without iron deficiency (Figure 1)—and the mean concentration of 25(OH)D was significantly (*p* = 0.000) lower in the group showing iron deficiency (Table 5).

Fluctuations in vitamin D due to latitude, weather pattern and length of solar exposure during summer are well known [10,28], but the role of iron in vitamin D metabolism is less clear. Iron, as a component of the cytochrome P450 monooxygenase superfamily, participates in synthesis of the active form of vitamin D_3_, not only in the last stage of its bioactivation from 25(OH)D to 1,25-dihydroxyvitamin D_3_ (25-hydroxyvitamin D 1-α-hydroxylase—CYP27B1), but also in the earlier stage in which cholecalciferol is converted to 25(OH)D (25-hydroxylase—CYP2R1) [38,53]. Therefore, as a consequence of iron deficiency, activity of these iron-containing enzymes may be lowered, and hence, a deficit in vitamin D_3_ may occur. This important role of iron in the synthesis of vitamin D was clearly confirmed by Katsumata et al. [39], who reported that dietary iron deficiency in rats caused diminished 1α-hydroxylase activity, leading to a decrease in serum 1,25-dihydroxyvitamin D_3_ concentration. The mentioned findings may explain why, in an earlier study, Heldenberg et al. [36] showed that a single intramuscular injection of iron in infants with iron deficiency anemia resulted in an increase in 25(OH)D concentration. These facts may also explain why, in the present study, the concentration of the metabolite, 25(OH)D, was significantly lower in subjects with iron deficiency. Some observational studies indicate that iron deficiency may be a significant predictor of vitamin D level [24,27,36]; however, there are some trial studies which showed that iron supplementation had no effect on 25(OH)D level [37,54]. Azizi-Soleiman [1] and Katsumata et al. [39] emphasize that the reason for this might be the degree of iron deficiency. So far, it is not known how severe iron deficiency must be to impair vitamin D synthesis, so we attempted to determine the value of ferritin concentration at which the risk of vitamin D deficiency starts to significantly increase. The ORs calculated for different values of ferritin (from 30 to 12 µg/L) indicate that the risk of vitamin D deficiency started to be significant at ferritin concentrations below 30 µg/L. The gradual increase in OR, from 1.75 (95% CI 1.02–2.99) at 30 µg/L ferritin to 4.60 (95% CI 1.81–11.65), in athletes with stage II ID and IDA suggests that disorders in vitamin D synthesis can arise along with worsening iron status.

The present study has some strengths but also some limitations. The strengths are as follows: the availability of data from healthy young athletes only, a relatively high number of subjects with iron and vitamin D deficiencies and the opportunity to perform a multivariable-adjusted logistic regression analysis, taking into account some confounders (age, season of blood collection, as well as in- and outdoor sports disciplines). Regarding limitations, first, the cross-sectional study design did not allow us to draw definite conclusions concerning causal relationships between studied nutrients. Another limitation is the lack of international, unequivocal threshold values for diagnosing both vitamin D and iron deficiency. Furthermore, as mentioned above, the lack of data on hepcidin and interleukins did not allow for a more accurate explanation of the presumed anti-inflammatory effect of vitamin D on iron status in physically active people. Lastly, we also lacked data on time spent outdoors (sun exposure), nutrient intake and vitamin D and iron supplementation, because the study was performed during periodic medical examinations, and it was impossible to perform interviews regarding supplementation.

## 5. Conclusions

To the best of our knowledge, this is the first study to analyze the mutual relationship between vitamin D and iron status among a large group of healthy, physically active females.

The current results clearly indicate an association between both analyzed nutrients. However, due to the observational study design, it is difficult to assess exactly which of these nutrients exerts a stronger influence on the other. It only seems that in healthy, physically active females the influence of iron deficiency on vitamin D status is greater. This is suggested by a higher percentage of vitamin D deficiency among subjects with poor iron status than vice versa, as well as a gradual increase in the risk of vitamin D deficiency, accompanied by progressive deterioration of iron status and the lack of such a relationship in the opposite direction.

Further rigorous, randomized controlled trials examining the effect of supplementation of both vitamin D and iron on specific biomarkers are needed to understand the exact mechanism underlying the mutual dependence of both nutrients.

Since deficits of iron and vitamin D are two common nutritional deficiencies and both nutrients interact with each other, it would be appropriate to monitor iron and vitamin D nutritional status simultaneously. Due to the impact of iron and vitamin D on health and on physical fitness, and because female athletes are a particularly high risk group for iron deficiency, a good status of both nutrients should especially concern them.

## Figures and Tables

**Table 1:** Characteristics of the studied population (mean ± standard deviation (SD)).

*n*	Age (Years)	Body Height (m)	Body Mass (kg)	Body Fat (%)	Athletic Experience (h/Week)
219 *	20.0 ± 4.4	1.74 ± 0.8	64.8 ± 7.5	23.3 ± 3.6	7.0 ± 3.4

* Canoe sprint: *n* = 40, cycling (road, track, and mountain bike): *n* = 27, handball: *n* = 19, rowing: *n* = 14, speed skating (long and short tracks): *n* = 21, taekwondo: *n* = 5, volleyball: *n* = 93.

**Table 2:** Indices of iron status, inflammatory markers and 25(OH)D concentrations in 219 female athletes (mean values, ±SD and ranges).

Variables	Units	Mean Values	Ranges	Reference Values
25(OH)D	nmol/L	74.8 ± 23.8	(10.8–132.3)	75–250
Ferritin	µg/L	34.8 ± 22.4	(2.7–135.2)	16–120
sTfR	mg/L	5.7 ± 2.0	(2.3–18.6)	2.9–8.3
TIBC	µmol/L	59.0 ± 6.8	(44.4–78.5)	44.6–69.6
Iron	µmol/L	15.2 ± 7.2	(1.97–45.8)	6.6–29.5
Hb	g/L	135 ± 6.9	(116–154)	120–160
RBC	×10^12^/L	4.6 ± 0.3	(3.6–5.4)	4.2–5.4
Hct	%	40.3 ± 2.0	(34.7–45.6)	37–47
MCH	pg	29.5 ± 1.4	(24.7–32.6)	26–32
MCV	fl	87.8 ± 3.7	(77–99)	81–99
CH	pg	29.4 ± 1.4	(23.7–32.4)	-
MCHC	g/L	336 ± 9.8	(312–377)	330–370
RDW	%	12.8 ± 0.61	(11.6–14.8)	11.5–14.5
RETIC	%	1.4 ± 0.34	(0.57–2.77)	0.5–2.5
#RETIC	10^9^/L	63.9 ± 15.22	(29–128)	22–139
MCVr	fl	101.4 ± 3.0	(90–111)	101–119
CHr	pg	31.4 ± 1.44	(26–34)	27–32
CHCMr	g/dL	31.0 ± 1.24	(27–35)	33–37
LowCHr	%	9.9 ± 10.0	(0.7–68.7)	-
LowCHm	%	24.0 ± 13.5	(6.0–81.8)	-
HYPOm	%	0.86 ± 1.31	(0.02–11.2)	-
HYPOr	%	13.0 ± 11.7	(0.6–67.4)	-
MICROm	%	0.65 ± 0.6	(0.1–4.86)	-
CRP	mg/L	0.37 ± 0.6	(0–4)	to 5
ESR	mm/h	4.4 ± 2.3	(1–13)	to 15
WBC	×10^9^/L	5.6 ± 1.2	(3.2–10.4)	4.5–10.4

25(OH)D—25-hydroxyvitamin D; sTfR—soluble transferrin receptor; TIBC—total iron binding capacity; Hb—hemoglobin concentration; Hct—hematocrit; MCH—mean corpuscular hemoglobin; RBC—red blood cell count; MCV—mean corpuscular volume; CH—mean cellular hemoglobin content in erythrocytes; MCHC—mean corpuscular hemoglobin concentration; RDW—red cell distribution width; RETIC—absolute reticulocyte count as a percentage; #RETIC—absolute number of reticulocytes; MCVr—mean corpuscular volume of reticulocytes; CHr—mean cellular hemoglobin in reticulocytes; CHCMr—mean cellular hemoglobin concentration in reticulocytes; LowCHr—percentage of red blood cells with decreased mean cellular hemoglobin content in reticulocytes; LowCHm—percentage of red blood cells with decreased mean cellular hemoglobin content in erythrocytes; HYPOm—percentage of red blood cells with decreased cellular hemoglobin concentration; HYPOr—percentage of reticulocytes with decreased cellular hemoglobin concentration; MICROm—percentage of microcytic erythrocytes; CRP—c-reactive protein concentration, ESR—erythrocyte sedimentation rate, WBC—white blood cell count.

**Table 3:** Odds ratios (ORs) with confidence intervals (95% CI) of vitamin D and iron deficiency, adjusted for additional factors using multivariate logistic regression analysis.

	OR	95% CI	*p*
Vitamin D deficiency			
Iron deficiency (ferritin < 16 µg/L)	2.96	1.45–6.02	0.003
Length of day *	2.29	1.28–4.07	0.005
Iron deficiency			
25(OH)D < 75 nmol/L	2.73	1.32–5.62	0.007
Age	0.82	0.73–0.91	0.000

* 0: April l–September (effective synthesis of vitamin D); 1: October l–March.

**Table 4:** Odds ratios (ORs) with confidence intervals (95% CI) of: (A) insufficient level of vitamin D in groups with various levels of ferritin as a criterion of stage I of iron deficiency and in subjects with more severe iron deficiency (stage II of ID and iron deficiency anemia); and (B) iron deficiency in subjects with 25(OH)D concentrations below 75 and 50 nmol/L.

		OR	95% CI	*p*
A	Vitamin D deficiency			
	Ferritin < 30 µg/L	1.75	1.02–2.99	0.040
	Ferritin < 16 µg/L	3.14	1.56–6.31	0.001
	Ferritin < 12 µg/L	3.56	1.60–7.91	0.002
	stage II of ID + IDA *	4.60	1.81–11.65	0.001
B	Iron deficiency			
	25(OH)D < 75 nmol/L	3.14	1.56–6.31	0.001
	25(OH)D < 50 nmol/L	3.18	1.09–9.26	0.030

* the subjects with latent iron deficiency and with iron deficiency anemia.

**Table 5:** Serum 25(OH)D concentration in female athletes with normal iron status and iron deficiency (mean ± SD).

	Normal Iron Status	Iron Deficiency	*p*
25(OH)D nmol/L	77.0 ± 19.0	65.0 ± 14.5	0.000

**Table 6:** Iron status and blood morphology indices in athletes with sufficient or insufficient (VDD) serum 25(OH)D concentrations (mean ± SD).

Variables	Units	Sufficient Vitamin D	Vitamin D Deficiency	*p*
Ferritin	µg/L	37.8 ± 24.0	31.4 ± 20.5	0.043
sTfR	mg/L	5.1 ± 1.6	6.1 ± 2.3	0.001
TIBC	µmol/L	57.6 ± 6.4	60.1 ± 8.1	0.016
Iron	µmol/L	16.7 ± 7.5	13.7 ± 6.6	0.004
Hb	g/L	135 ± 6.7	136 ±7.1	0.39
Hct	%	40.1 ± 2.0	40.3 ± 2.0	0.33
MCH	pg	29.7 ± 1.4	29.4 ± 1.5	0.22
RBC	×10^12^/L	4.56 ± 0.26	4.64 ± 0.27	0.029
MCV	fl	88.2 ± 3.6	87.4 ± 3.7	0.22
CH	pg	29.6 ± 1.4	29.3 ± 1.4	0.08
MCHC	g/L	337 ± 10	337 ± 10	0.90
RDW	%	12.8 ± 0.5	12.8 ± 0.7	0.56
RETIC	%	1.37 ± 0.34	1.42 ± 0.33	0.34
#RETIC	×10^9^/L	62.3 ± 15.1	65.5 ± 15.0	0.17
MCVr	fl	102 ± 3	101 ± 3	0.020
CHr	pg	31.6 ± 1.5	31.2 ± 1.4	0.049
CHCMr	g/dL	31.0 ± 1.2	31.0 ± 1.3	0.84
LowCHr	%	9.18 ± 9.44	10.48 ± 10.36	0.17
LowCHm	%	22.8 ± 12.6	24.9 ± 13.6	0.22
HYPOm	%	0.77 ± 1.08	0.93 ± 1.47	0.37
HYPOr	%	12.8 ± 11.5	13.1 ± 11.8	0.97
MICROm	%	0.57 ± 0.44	0.73 ± 0.69	0.10
WBC	×10^9^/L	5.48 ± 1.18	5.77 ± 1.28	0.08

sTfR—soluble transferrin receptor; TIBC—total iron binding capacity; Hb—hemoglobin concentration; Hct—hematocrit; MCH—mean corpuscular hemoglobin; RBC—red blood cell count; MCV—mean corpuscular volume; CH—mean cellular hemoglobin content in erythrocytes; MCHC—mean corpuscular hemoglobin concentration; RDW—red cell distribution width; RETIC—absolute reticulocyte count as a percentage; #RETIC—absolute number of reticulocytes; MCVr—mean corpuscular volume of reticulocytes; CHr—mean cellular hemoglobin in reticulocytes; CHCMr—mean cellular hemoglobin concentration in reticulocytes; LowCHr—percentage of red blood cells with decreased mean cellular hemoglobin content in reticulocytes; LowCHm—percentage of red blood cells with decreased mean cellular hemoglobin content in erythrocytes; HYPOm—percentage of red blood cells with decreased cellular hemoglobin concentration; HYPOr—percentage of reticulocytes with decreased cellular hemoglobin concentration; MICROm—percentage of microcytic erythrocytes; WBC—white blood cell count.

## Supplementary Materials

Supplementary File 1
